# Supplementary material for: ProteinShader: illustrative rendering of macromolecules
Source: BMC Struct Biol. 2009 Mar 30;9:19. doi: 10.1186/1472-6807-9-19 (PMC2672931; doi:10.1186/1472-6807-9-19)
Supplement: Additional file 1 — ProteinShader program without source code. This compressed file contains the complete ProteinShader program including associated libraries, but no source code. A README.txt file gives an overview of the ProteinShader distribution, and the index.html file in the help subdirectory has directions on getting started with the program as well as a set of tutorials. [file 1472-6807-9-19-S1.zip › ProteinShader-beta-0_9_4-binary/help/api/org/proteinshader/graphics/typography/GlyphSetFactory.html]

GlyphSetFactory (ProteinShader API)


|  |  |  |
| --- | --- | --- |
| **Overview** \| **Package** \| **Class** \| **Use** \| **Tree** \| **Deprecated** \| **Index** \| **Help** | |  |
| **PREV CLASS**   **NEXT CLASS** | **FRAMES**    **NO FRAMES**     **All Classes** |  |
| SUMMARY: NESTED \| FIELD \| CONSTR \| METHOD | DETAIL: FIELD \| CONSTR \| METHOD |  |


---


## org.proteinshader.graphics.typography Interface GlyphSetFactory

**All Known Implementing Classes:**: GLF2GlyphSetFactory

---

``` public interface GlyphSetFactory ```

Defines the interface for a factory that can read glyphs from a source
and produce a GlyphSet object.

---

| **Method Summary** | |
| --- | --- |
| `GlyphSet` | `readGlyphSet(Properties p)`             Reads information from a source and constructs a GlyphSet object. |
| `void` | `setDebug(boolean debug)`             Setting debug to true will activate several print statements that are useful for troubleshooting. |

**Method Detail**

### readGlyphSet

```
GlyphSet readGlyphSet(Properties p)
                      throws GlyphException
```

:   Reads information from a source and constructs a GlyphSet object.

---


### setDebug

```
void setDebug(boolean debug)
```

:   Setting debug to true will activate several print statements that
    are useful for troubleshooting.

    :   **Parameters:**: `debug` - boolean value to turn debugging on or off.


---


|  |  |  |
| --- | --- | --- |
| **Overview** \| **Package** \| **Class** \| **Use** \| **Tree** \| **Deprecated** \| **Index** \| **Help** | |  |
| **PREV CLASS**   **NEXT CLASS** | **FRAMES**    **NO FRAMES**     **All Classes** |  |
| SUMMARY: NESTED \| FIELD \| CONSTR \| METHOD | DETAIL: FIELD \| CONSTR \| METHOD |  |


---

# *Copyright © 2007-2008*
